# Histopathological Features of Myxoid Pleomorphic Liposarcoma in an African Pygmy Hedgehog (*Atelerix Albiventris*)

**DOI:** 10.3390/vetsci9110642

**Published:** 2022-11-19

**Authors:** Eun-Joo Lee, Kyu-Shik Jeong

**Affiliations:** Department of Veterinary Pathology, College of Veterinary Medicine, Kyungpook National University, Daegu City 41566, Republic of Korea

**Keywords:** myxoid pleomorphic liposarcoma, pleomorphic liposarcoma, myxoid liposarcoma, malignant fibrous histiocytoma, African pygmy hedgehog

## Abstract

**Simple Summary:**

Liposarcoma is a malignant tumor that is derived from fat tissue. Liposarcoma is one of the most common malignant tumors in humans and animals. Liposarcomas are classified into six groups according to the WHO: dedifferentiated liposarcoma, myxoid liposarcoma, pleomorphic liposarcoma, liposarcoma not otherwise specified, atypical spindle cell/pleomorphic lipomatous and myxoid pleomorphic liposarcoma. Each subtype shows distinct pathological features, responses to chemotherapy and/or radiotherapy, and clinical outcomes. In this case, a myxoid pleomorphic liposarcoma was observed in a 5-year-old male African pygmy hedgehog. Myxoid pleomorphic liposarcoma is characterized by pathological features of both pleomorphic liposarcoma and myxoid liposarcoma. The tumor consisted of ~60% of the myxoid substance area and ~30% of pleomorphic neoplastic cells. The tumor presented similar clinical outcomes, including metastasis and prognosis, to myxoid liposarcoma rather than pleomorphic liposarcoma. This suggests that analyzing the proportion of pleomorphic area and myxoid area in myxoid liposarcoma could provide important information for predicting clinical outcomes.

**Abstract:**

Myxoid pleomorphic liposarcoma is characterized by pathological features of both pleomorphic liposarcoma and myxoid liposarcoma, as the name suggests. In this case, a myxoid pleomorphic liposarcoma was observed in a 5-year-old male African pygmy hedgehog. It consisted of ~60% of the myxoid substance area with proliferating round cells and ~30% of pleomorphic neoplastic cells. The subject presented with extrapulmonary metastasis, but a good prognosis during 6 months of follow-up, which is similar to the characteristics of myxoid liposarcoma. The histopathological features of myxoid pleomorphic liposarcoma may reflect the features of either myxoid liposarcoma or pleomorphic liposarcoma depending on the proportion of each histopathological feature. The proportion of the pleomorphic area and the myxoid area may offer information on the prognosis and metastasis of myxoid pleomorphic liposarcoma, which will be helpful for setting up a treatment plan. Thus, analyzing the proportion of pleomorphic area and myxoid area could be suggested as one of the ways to predict clinical outcomes. In addition to the fact that this is the first case of a myxoid pleomorphic liposarcoma in hedgehogs, this case is meaningful, considering the unique histopathological characteristics and rare incidence of myxoid pleomorphic liposarcoma that could be important in humans as well.

## 1. Introduction

Liposarcoma is one of the soft tissue sarcomas and is a malignant tumor that is derived from adipose tissue and composed of adipoblasts [1]. Liposarcoma is one of the most common malignant tumors, accounting for 12.8% of soft-tissue tumors in humans [2]. However, unlike in humans, liposarcoma is not common in animals, accounting for 0.2–0.5% in canine tumors [1]. Liposarcomas have been classified into four subtypes according to the World Health Organization (WHO) classification until 2020: dedifferentiated liposarcoma, myxoid liposarcoma, pleomorphic liposarcoma, and liposarcoma not otherwise specified [3]. Dedifferentiated liposarcoma is characterized by poorly differentiated adipoblasts. Myxoid liposarcoma presents abundant myxoid stroma with immature adipoblasts. Pleomorphic liposarcoma is composed of neoplastic cells which show pleomorphism with multinucleated giant cells. The neoplastic adipoblasts which have intracytoplasmic lipid vacuoles account for only a small portion of the total tumor of pleomorphic liposarcoma. Liposarcoma not otherwise specified indicates a mixture form of the subtypes of liposarcoma which cannot be classified into any of the other three subtypes [4]. In early 2020, the WHO classification of Tumors of Soft Tissue and Bone was updated and the fifth edition of the classification was published, which includes myxoid pleomorphic liposarcoma (MPL) and atypical spindle cell/pleomorphic lipomatous tumor as new classification categories in addition to the previous WHO classification, considering the clinical importance and incidence [5,6]. MPL is a mixed form of pleomorphic liposarcoma and myxoid liposarcoma. Myxoid pleomorphic liposarcoma is known for its early onsets, such as in adolescence or young adults, while other liposarcoma subtypes occur in middle-aged to old adults [4]. Atypical spindle cell/pleomorphic lipomatous tumor is a benign tumor showing an infiltrative margin with various spindle cells, adipocytes, and adipoblasts [5,6].

In this study, we report MPL in an African pygmy hedgehog which presents histopathological characterization of both pleomorphic liposarcoma and myxoid liposarcoma. Considering the lack of reports of MPL in human and veterinary fields, it would be meaningful to deal with a detailed description of histopathological features, differential diagnosis, and prognosis. 

## 2. Materials and Methods

The specimens were submitted to the laboratory of veterinary pathology of the Kyungpook National University for histopathological examination. The tissues were fixed in neutral buffered 10% formalin for two days. The fixed tissues were processed routinely and embedded in paraffin wax. The paraffin tissue block was cut into 6 μm thickness. Hematoxylin and Eosin (H&E) staining was performed. Immunohistochemistry staining was routinely performed. Briefly, antigens were retrieved by incubating in pre-heated 0.01 M citrate buffer for 30 min. Then, the slides were cooled down for 1 h at room temperature. The slides were incubated with blocking buffer, followed by incubation with primary antibodies including vimentin (SC-6260, 1:200), alpha-smooth muscle actin (SC-53142, dilution 1:200), CD68 (SC-20060, dilution 1:100), desmin (SC-23879, dilution 1:200), and S100 (SC-53438, dilution 1:100) (Santa Cruz, Dallas, TX, USA). The antibodies were detected using avidin–biotin complex (ABC kit) and visualized using DAB at room temperature. Leica digital camera 3-4.9 (Leica camera, Wetzlar, German) was used for capturing pictures of gross examination. The Leica Application Suite program (version 2.5.0 R1; Leica Microsystem, Wetzlar, German) was used for imaging microscopic images and analyzing the area of myxoid substance and pleomorphic area.

## 3. Case Presentation

**Figure 1:**
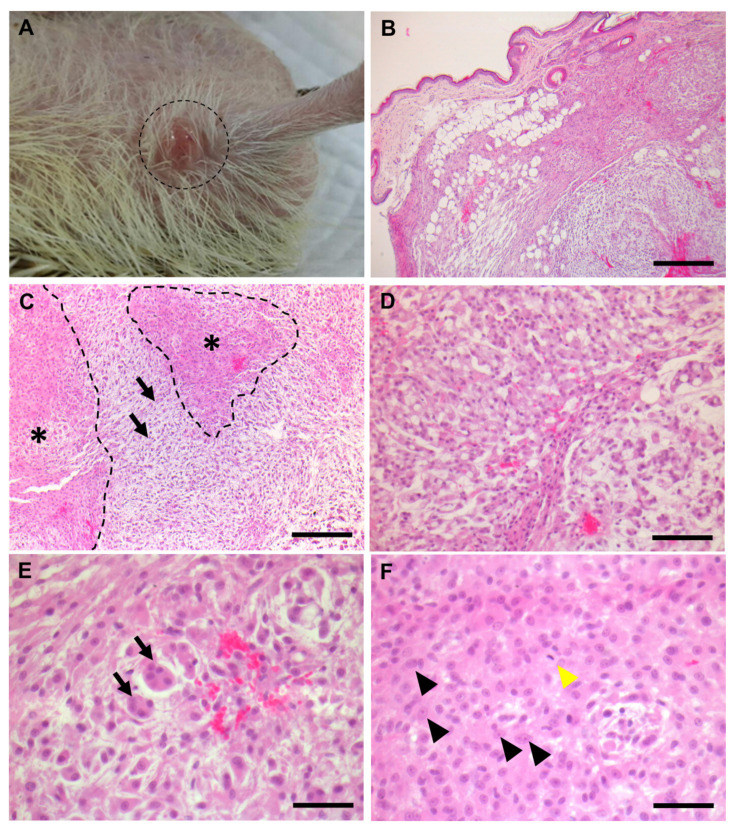
**Pathological observation of the pleomorphic myxoid liposarcoma.** (**A**) Gross observation with gross pathology. The neoformations were protrude and dome-shaped. (**B**) Microscopic observations of low magnification. The neoformations were located in the subcutaneous layer of the right abdomen. The neoformations were not encapsulated. Scale bar = 500 μm. (**C**–**F**) Microscopic observations of high magnification. (**C**) The neoformations were composed of the myxoid proliferation area (black arrows) and cellular area (outlined and marked with asterisks). The myxoid proliferation area is composed of proliferating stellate cells and abundant myxoid materials. Scale bar = 200 μm. (**D**) Neoplastic adipoblasts with variable size of intracytoplasmic clear vacuoles. Scale bar = 100 μm. (**E**) Large bizarre multinucleated giant cells (arrows). Scale bar = 50 μm. (**F**) Mitotic figures were sometimes observed (yellow arrowhead). Epithelioid cells were observed (black arrowheads). The epithelioid cells had abundant eosinophilic cytoplasm with round-to-oval-shaped nuclei. One to two prominent nucleoli were observed in the nucleus. Scale bar = 50 μm.

A 5-year-old male hedgehog had neoformations on the skin of the *regio abdominis caudalis* (Figure 1A) and lower extremities. The sizes of the neoformations rapidly increased within a month, which were 1.0 × 1.0 × 0.5 cm and 1.0 × 0.7 × 0.5 cm, respectively, when the specimens were submitted to the laboratory of veterinary pathology of the Kyungpook National University. At the gross pathologic examination, the neoformations were slightly greasy and firm. The color of the cut surfaces of the tumors were white to ivory. The neoformations were located in the subcutaneous layer and were not encapsulated. (Figure 1B). It was hard to find clear demarcation of the neoformations.

On histopathological findings, the neoformations displayed typical characteristics of either myxoid liposarcoma or pleomorphic liposarcoma. The neoformations were mainly composed of two distinct areas; one with abundant myxoid substances and the other with high cellularity with pleomorphic cells (Figure 1C,D). The area with myxoid substance accounted for around 60% of the total area, whereas the area with high cellularity occupied 30% of the total area. The area with myxoid substance also demonstrated stellate mesenchymal cells with storiform arrangement. However, it was hard to find collagenous stroma. There were neoplastic adipoblasts with various sizes of intracytoplasmic lipid vacuoles accounted for approximately 10% of the total area of the neoformations (Figure 1C,D). Notably, bizarre multinucleated giant cells were occasionally observed (Figure 1E). In a small part of the neoformations, round-to-oval-shaped histiocyte-like cells were observed, which is similar to malignant fibrous histiocytoma (Figure 1E). The histiocyte-like cells were round-to-polyhedral in shape with eosinophilic cytoplasm. Additionally, the nuclei of the neoplastic cells were large and round-to-oval-shaped with one to two prominent nucleoli. Pleomorphism was remarkable in both the cytoplasm and nucleus of the neoplastic cells. Mitotic figures were occasionally observed (Figure 1F). In some parts of the neoformations, a “herringbone pattern” was observed, which is one of the characteristics of spindle cell tumors. Neovascularization was not prominent in the neoformations.

**Figure 2:**
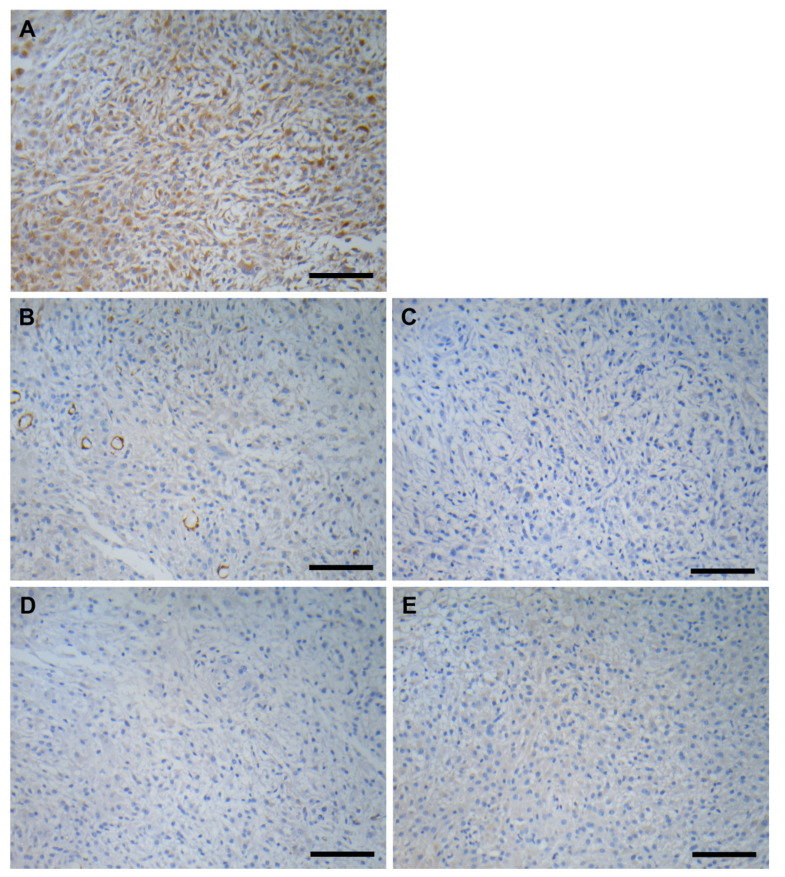
**Immunohistochemistry of pleomorphic myxoid liposarcoma.** The neoplastic cells were positive for (**A**) vimentin, and negative against (**B**) alpha-smooth muscle actin, (**C**) CD68, (**D**) desmin, and (**E**) S100. Scale bar = 200 μm.

Differential diagnosis was performed on the immunohistochemistry stainings using the following antibodies: alpha smooth muscle actin, CD68, desmin, S100 and vimentin (Figure 2). Around 90% of neoplastic cells presented strong immunoreactivity to vimentin, whereas the neoplastic cells were negative for alpha-smooth muscle actin, CD68, desmin, and S100, which implies little possibility of malignant fibrous histiocytoma, malignant Schwannoma, and rhabdomyosarcoma diagnosis. Considering the histopathological findings and the results of immunohistochemistry, the neoformations were diagnosed as myxoid pleomorphic liposarcoma. 

## 4. Discussion

The classification of liposarcoma was updated in 2002, 2013, and 2020 [3,5,6]. One of the reasons for the frequent updates of the classification could be the high incidence of liposarcoma, which accounts for 12.8% of sarcoma in humans [2]. Additionally, it might be due to the diverse cell populations of liposarcoma, which include adipoblasts, stellate cells, and round cells, and pleomorphism of neoplastic cells, which make subtypes more complicated. MPL has been added to the liposarcoma subclassification as a new subtype in 2020 to reflect the clinical importance and increasing incidence of pleomorphic liposarcoma with myxoid substance and myxoid liposarcoma with pleomorphism [5,6]. 

It is necessary to make an exact diagnosis of liposarcoma subtypes rather than simply diagnosing liposarcoma without subclassification. The prognosis and treatment of choice of liposarcoma are dependent on the subtypes, which emphasizes the importance of the exact diagnosis of the subtypes of liposarcoma. In humans, each subtype presents different sensitivity to chemotherapy and radiotherapy. For example, 38% of patients with myxoid liposarcoma respond to doxorubicin and/or ifosfamide as a first-line chemotherapy, whereas the patients with well-differentiated or dedifferentiated liposarcoma present an 11% response rate [7]. Consistent with the different chemosensitivity depending on the subtypes of liposarcoma, the progression-free survival rate after chemotherapy is also different depending on the subtypes of liposarcoma [7]; not only for the chemotherapy, but also the sensitivity to the radiotherapy was different depending on subtypes. For example, myxoid liposarcoma was remarkably sensitive to radiotherapy when compared to other subtypes of liposarcoma in humans [8].

Unfortunately, there were not enough reports to show the relevance of the biological behaviours of each subtype in animals. This could be due to the low incidence of liposarcoma in animals which do not have enough cases to classify, although there is a report that chemotherapy after surgical excision on the pleomorphic liposarcoma was not sufficient to prevent metastasis and recurrence [9]. Considering the importance of subtypes of liposarcoma in humans, making an exact diagnosis of a subtype of liposarcoma might be important in animals for setting a plan of treatment modules and anticipating the progesssion of the liposarcoma. However, more case reports are required in the veterinary field with detailed information of biological behaviour and subclassification of liposarcoma to reach the conclusion that liposarcoma in animals have the same characteristics as in humans. Despite the importance of the exact diagnosis of subtypes of liposarcoma in humans, the information on histological features, prognosis, and metastasis of MPL is lacking compared to other subtypes of liposarcoma, even in humans. There are only a few reports of MPL because it was added to the WHO classification recently, and the incidence of MPL is also rare [5,6]. Although pleomorphic liposarcoma with myxoid proliferation has been reported, only limited histological descriptions are available in humans [10]. Unlike in humans, liposarcoma is not common in animals and many of them are unclassified [11]. In particular, MPL is extremely rare in the field of veterinary medicine. This is the first case of MPL in the veterinary field based on a search using the key words “animal”, “myxoid pleomorphic liposarcoma”, and “veterinary” on PUBMED for the period from 1989 to 2022. This could be due to the low incidence of MPL in animals. There is a possibility that MPL could have been considered as myxoid liposarcoma with high pleomorphism or pleomorphic liposarcoma with myxoid substance since the subclassification of liposarcoma was recently updated. In 2016, Plumlee et al. reported a case of high-grade myxoid liposarcoma in a dog which had both characteristics of pleomorphic liposarcoma and myxoid liposarcoma [12].

Considering the importance of the exact diagnosis of subtypes of liposarcoma as mentioned above and the rarity of MPL in veterinary medicine, the case description including histopathological characteristics, metastasis, relapse, and prognosis of MPL is worth reporting. Here, we report a myxoid pleomorphic liposarcoma in a hedgehog, which presented histological features of both myxoid liposarcoma and pleomorphic liposarcoma. 

Since pleomorphism and myxoid proliferation are the general histological characteristics of sarcoma, pathologists are recommended to pay particular attention to diagnosing tumors that are characterized by remarkable pleomorphism and myxoid substances. The differential diagnosis of MPL includes myxoid malignant fibrous histiocytoma, myxoid liposarcoma, and pleomorphic liposarcoma due to histopathological similarity. First, MPL can be misdiagnosed as myxoid malignant fibrous histiocytoma since it presents multinucleated giant cells with myxoid substance, which are the same histological features of MPL. Additionally, MPL has only a small portion of intracytoplasmic lipid vacuoles, which is one of the characteristics of malignant fibrous histiocytoma, leading to confusion with MPL. Additionally, myxoid malignant fibrous histiocytoma is difficult to distinguish by IHC from myxoid pleomorphic liposarcoma, since they share the same immunohistochemical reactivity [13]. Unlike myxoid malignant fibrous histiocytoma, however, MPL does not have collagenous stroma and storiform spindle cells, so MPL can be differentiated from myxoid malignant fibrous histiocytoma [14]. In this case, a small number of adipoblasts with lipid vacuoles was observed, which was around 10% of the total area, but the collagenous matrix and spindle cells were hardly observed, so the myxoid malignant fibrous histiocytoma was ruled out of the differential diagnosis. Based on these histopathological features, the present neoformations were diagnosed as myxoid pleomorphic liposarcoma. 

The percentage of pleomorphic area and myxoid area in MPL might have a relevance to clinical outcomes, including metastasis and prognosis. MPL presents histopathological features of both myxoid liposarcoma and pleomorphic liposarcoma. MPL, which has a greater myxoid area compared to pleomorphic area, tends to present characteristics of myxoid liposarcoma such as extrapulmonary metastasis and good prognosis. On the other hand, MPL with a large pleomorphic area might show the characteristics of pleomorphic liposarcoma, such as pulmonary metastasis and poor prognosis. The proportion of specific cell populations in tumors could have relevance to clinical outcomes. Even in the same subtype, the prognosis can be different depending on the percentage of each cell population. In myxoid liposarcoma, for example, the higher percentage of round cells (>5%) indicates worse prognosis with metastasis, which make it challenging to predict the prognosis without an exact diagnosis and detailed histopathological description [15,16].

This case presents a higher percentage of myxoid proliferation compared to the pleomorphic area. The features of clinical outcomes of this case are similar to myxoid liposarcoma. Regarding the metastasis, the primary tumor metastasized to the lower extremities, but it did not present pulmonary metastasis during the 6 months of follow-up. The percentage of pleomorphic area and myxoid area in MPL might be also related to the preference of extrapulmonary and pulmonary metastasis, respectively. Myxoid liposarcoma and pleomorphic liposarcoma present distinct features of metastasis; myxoid liposarcoma have a preference for extrapulmonary metastasis [17,18], whereas 70% of pleomorphic liposarcoma tends to metastasize to the lung [19]. A greater proportion of pleomorphic population might increase the likelihood that MPL will have pulmonary metastasis, which is a characteristic of pleomorphic liposarcoma. On the other hand, when the myxoid area occupies a large portion, the possibility of extrapulmonary metastasis, which is a characteristic of myxoid liposarcoma, might increase [20]. In addition, the predominance of either the myxoid area or pleomorphic area might be related to the prognosis. Myxoid liposarcoma presents a relatively good prognosis, whereas pleomorphic liposarcoma shows poor prognosis of 30~40% survival rate after 5 years [21,22]. This case presents a good prognosis, suggesting similar features to myxoid liposarcoma. Thus, the histopathological similarity to either myxoid liposarcoma or pleomorphic liposarcoma might help to predict the prognosis of MPL. Thus, the characteristics of MPL, including metastasis and prognosis, might be similar to either myxoid liposarcoma or pleomorphic liposarcoma, depending on how much area is occupied by either the myxoid area or pleomorphic area. 

MPL usually occurs in young adults [23]. In this case, however, MPL was observed in an older adult, considering that the lifespan of a companion hedgehog is 5 to 7 years. This case suggests the possible occurrence of MPL in the middle-to-old-aged population, although it could be considered as a species-dependent difference. In general, histopathological features of tumors are shared between species, making human sarcoma classification applicable to the field of veterinary medicine. This is the first case of MPL in an African pygmy hedgehog, although there was a report of well-differentiated liposarcoma in a hedgehog, which is the most common subtype of liposarcoma in humans [24,25]. There is no reported case of MPL in hedgehogs, although the number of reports of tumors in hedgehogs has been increasing due to the remarkably increase in the number of hedgehogs as companion animals [25,26]. 

However, the importance of this case is more related to the histopathological description and metastasis, in addition to this being the first known case of MPL in hedgehogs. Besides the fact that this is the first known case of MPL in hedgehogs, this case is worth reporting considering the unique histopathological characteristics and rare incidence of MPL, which could also be meaningful information for humans as well. 

## 5. Conclusions

This is the first case report of myxoid pleomorphic liposarcoma in veterinary medicine. The MPL presented both features of myxoid liposarcoma and pleomorphic liposarcoma. The tumor consisted of ~30% of myxoid substance and ~60% of pleomorphic neoplastic cells. The subject presented extrapulmonary metastasis, but relatively good prognosis, which are the features of myxoid liposarcoma. The proportion of the pleomorphic area and the myxoid area might be correlated to the prognosis and metastasis of MPL. Thus, analyzing the proportion of each cell population in MPL could be suggested to anticipate the biological behaviour of MPL.
